# Numerical Study of Bacteria Containing Droplets Aerosolized From Hot Surfaces

**DOI:** 10.1038/s41598-020-66081-y

**Published:** 2020-06-04

**Authors:** Ekaterina Mirskaya, Vladimir Maksimenko, Valery Zagaynov, Igor Agranovski

**Affiliations:** 1 School of Engineering and Built Environment, Griffith University, Nathan 4111, Queensland, Australia; 2 National Research Nuclear University (Moscow Engineering Physics Institute), 31, Kashirskoe shosse, 115409 Moscow, Russia

**Keywords:** Air microbiology, Natural hazards

## Abstract

The process of water droplet interaction with hot surface can result in droplet shooting off the surface. When the water is contaminated with bacteria the interaction causes substantial ambient air contamination due to aerosolization of live or injured microorganisms. This study investigates the behaviour of water droplets interacting with heated surfaces in the film boiling regime. A suggested mathematical model considers droplet shooting off conditions and following airborne droplet evolution due to cooling. The critical size of the droplet capable of taking off was modelled as a function of the wall temperature and droplet size. Following the departure from the hot surface, droplet cooling time mainly depends on the initial droplet radius while the influence of the ambient temperature is marginal. The experimental part of the study was focused on (1) investigation of the size of droplets capable of departing from the hot surface, and (2) evaluation of the influence of cooling time on the survivability of two common environmental bacterial species, Gram-negative *Escherichia coli* and Gram-positive *Bacillus subtilis*. Droplets with the sizes of up to one millimetre shooting off the hot surfaces were detected, which correlates with the theoretical results. It was found that, under realistic physical conditions, the process of liquid interaction with hot surface does not ensure an efficient microbial inactivation. It was also shown that the shortest cooling time was associated with higher survival rates of both bacterial strains used in this study. However, even for the longest cooling time of 15 seconds the amount of live bacteria in the aerosolized droplet carrier can be substantially high with recovery rates of approximately 50% for *B. subtilis*.

## Introduction

The process of interaction between a water droplet and hot surfaces has been widely investigated. A variety of the process parameters such as droplet dynamics, secondary droplet size, heat transfer and cooling effectiveness^1–3^ have been studied and described. Numerical simulations have been developed in order to model the impact of a droplet onto a hot surface, predict the secondary droplet size and investigate the cooling effectiveness^4–6^. The main parameters influencing the process are surface thermal properties and roughness^7^, an angle of impingement^1^, and a droplet diameter and velocity^8^. However, surface temperature remains the most important factor affecting the process of water-surface interaction^9^.

When the surface is superheated above the Leidenfrost temperature^6^,the direct contact between the heated wall and deposited droplet is limited due to immediate formation of vapour layer. This phenomenon refers to the film-boiling regime. Droplet hydrodynamics depend on the droplet size and impact velocity and may include droplet spread, rebound or disintegration^10^.

In our previous investigation we experimentally demonstrated that the water-surface interaction may be associated with generation of viable bacterial aerosols when the water is contaminated with bacteria^11^. It was shown experimentally, that the film boiling regime at the surface temperature range 330–380 °C results in the most efficient droplet rebound, splashing, and secondary droplet formation, and provided the highest rate of viable bacteria recovery in droplets produced as the result of interaction with the hot surface. Aerosolization of live microorganisms in liquid carriers resulting from interaction of microbial suspension with hot surfaces can potentially cause substantial contamination of ambient air in industrial (cooling of hot equipment, using of metal-working fluids in machining operations, etc) and residential (using of potentially microbially contaminated liquids to pour onto rocks in saunas, extinguishing BBQ equipment, etc.) environments. Considering that such process can potentially result in aerosolization of pathogenic bacteria, the mechanism requires special attention.

The main focus of this study was to develop a numerical model of the earlier demonstrated mechanism of water-surface interaction with the following experimental verification of theoretical outcomes. The model aimed to predict the size of a water droplet capable of departing from the hot surface and the rate of droplet cooling based on the ambient air parameters. An influence of the droplet cooling rate on bacterial survival is also experimentally investigated in this study.

## Methodology

### Mathematical model and numerical method

#### Droplet shooting off model

**Figure 1:**
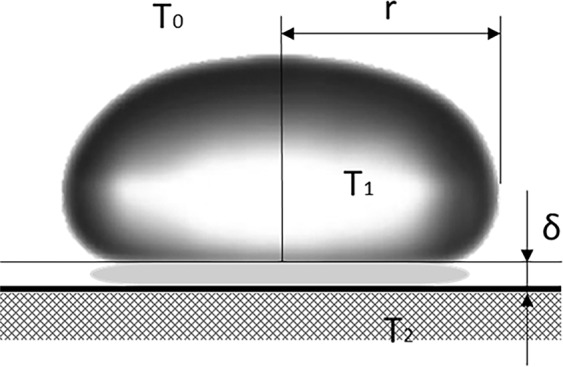
Water droplet resting on a vapour cushion.

A droplet is deposited on a heated surface in the film-boiling regime with low initial velocity. Figure 1 shows a water droplet “resting” on a vapour cushion. The droplet is nearly spherical, except it is flattened at the bottom^12^. The bottom surface of the droplet is a circle with the radius r. The droplet temperature is known to be 99 ± 1 °C^12^.

Heat balance equation is written in the form:

1 $$-\kappa {\nabla }T\pi {r}^{2}dt=\lambda dm$$

where *dm* is the mass of water evaporated during the time *dt*, *r* is the droplet radius, *k* is the thermal conductivity of water vapour, λ is the latent heat of vaporisation, and $\nabla T$ is the temperature gradient in the gap between the droplet and the wall which is defined as:

2 $${\nabla }T=\frac{{T}_{2}-{T}_{1}}{\delta }$$

where *T*_1_ and *T*_2_ are the droplet bottom and wall temperature respectively and *δ* is the thickness of the vapour layer.

The Meshchersky’s equation^13^ given below is a basic differential equation in the mechanics of variable-mass particles.

3 $$m\frac{dv}{dt}=-u\frac{dm}{dt}-mg$$

where *m* is the droplet mass and *u* is the relative velocity of water vapour to water droplet.

The equation was solved to capture the velocity of the droplet, where *m*_0_ is an initial droplet mass, *m(t)* is a droplet mass at time *t*:

4 $$v=u\,ln\,\frac{{m}_{0}}{m(t)}-gt$$

The droplet is supported up on the surface by the reactive power established due to evaporation of the droplet through the flat bottom surface. The mass *dm* carried away during the time *dt* can be expressed as follows:

5 $$dm={\rho }_{v}u\pi {r}^{2}dt$$

where *ρ*_*v*_ is the water vapour density.

From comparison of (1) and (5) it follows that the relative velocity of water vapour to water droplet is a constant which is defined as:

6 $${\rm{u}}=\frac{1}{{\rho }_{v}}\frac{\kappa ({T}_{2}-{T}_{1})}{\delta \lambda }$$

where *λ* is the latent heat of vaporisation of water.

While the droplet mass is calculated as:

7 $$m=4\pi {\rho }_{l}{r}^{3}/3$$

The evaporation rate is defined as:

8 $$\frac{dm}{dt}=4\pi {\rho }_{l}{r}^{2}\frac{dr}{dt}$$

where *ρ*_*l*_ is the density of water.

By rewriting Eq. (1)

9 $$\frac{dm}{dt}=-\frac{\kappa {\nabla }T\pi {r}^{2}}{\lambda }$$

Therefore,

10 $$\frac{dr}{dt}=-\frac{\kappa {\nabla }T}{4\lambda {\rho }_{l}}$$

And

11 $$r={r}_{0}-\frac{\kappa {\nabla }T}{4\lambda {\rho }_{l}}t$$

where *r*_*o*_ is the initial droplet radius.

By rewriting Eq. (11) in (4) and taking into account (7), the droplet velocity can be defined as follows:

12 $$v=-\,3u\,\mathrm{ln}\left(1-\frac{k\nabla T}{{4}_{l}}\frac{t}{{r}_{0}}\right)-gt$$

#### Critical radius of the droplet

The conditions when the droplet is not capable to leave the hot surface can be determined. Considering the basic situation when the droplet shoots off immediately, the travel distance is defined as:

13 $$h={\int }_{0}^{{t}^{\ast }}v(t)dt=\frac{3u}{\alpha }((1-\alpha {t}^{\ast })(ln(1-\alpha {t}^{\ast })-1)+1)-\frac{g{(t\ast )}^{2}}{2}=\frac{3u}{\alpha }-\frac{g{(t\ast )}^{2}}{2}$$

where

14 $$\alpha =\frac{\kappa {\nabla }T}{4\lambda {\rho }_{l}{r}_{0}}$$

and t^*^ is the droplet lifetime determined from Eq. (11):

15 $$0={r}_{0}-\frac{\kappa {\nabla }T}{4\lambda {\rho }_{l}}{t}^{\ast }$$

If the droplet does not take off immediately then the time τ required for the droplet to finally leave the surface is calculated from the assumption v(τ) = 0 and it is the solution of the equation:

16 $$\tau =-\frac{3u}{g}\,\mathrm{ln}\left(1-\frac{\kappa {\nabla }T}{4\lambda {\rho }_{l}}\frac{\tau }{{r}_{0}}\right)$$

The equation can be solved numerically. Then the distance travelled is:

17 $$h={\int }_{\tau }^{{t}^{\ast }}v(t)dt$$

#### Droplet evaporation during cooling

It is suggested that the droplet cools as it evaporates. The amount of heat is defined as:

18 $$mc({T}_{1}-{T}_{0})=\lambda \delta m,$$

where *c* is specific heat of water. For the purposes of this study the temperature of 40 °C is considered as the optimal temperature for bacteria to survive and remain viable. Therefore, the difference between the initial temperature of the water droplet on the heated surface and the safe temperature of 40 °C is 60 °C.

According to Fuchs^14^, the mass evaporated during a certain period of time is:

19 $$\delta m=It$$

where I is the rate of evaporation due to diffusion which is defined as:

20 $$I=\frac{4{r}_{0}}{R{T}_{0}}D({p}_{0}-{p}_{\infty })$$

where *µ* is the molecular weight of water, *p*_0_ is the vapour pressure in the vicinity to the droplet, *p*_*∞*_ is the vapor pressure at a large distance from the droplet, *D* is the diffusion coefficient and *R* is the ideal gas constant.

The time required for cooling down to the safe temperature is:

21 $$t=\frac{c{r}_{0}^{2}{\rho }_{l}(60)R{T}_{0}}{3\lambda {p}_{0}D}=0.018{r}^{2}{T}_{o}$$

### Experimental Investigation

#### Investigation of droplet size

A series of experiments were undertaken to investigate the size of the droplets departing from the hot surface. The experimental setup was similar to the one described in detail in [11]. In brief, a heating element with controllable temperature (Model PC-420D, Labnet International Inc., Edison, NJ, USA) was used for providing the required surface temperature of up to 500 °C. To avoid the escape of levitating droplets during the experiments and to ensure a uniform temperature distribution across the surface, the area of water-surface interaction was restricted by a stainless steel ring (diameter − 10 cm, height − 2 cm), centrally placed on the heated surface. The temperature of the plate was monitored by a calibrated K-type thermocouple operated by a digital dual input thermometer (Model TM-1300K, Protek, Allendale, NJ, USA). The heating element was strategically located to ensure that the vast majority of potentially released droplets will be passing through a measuring laser system of the Spraytec laser diffraction instrument (Malvern Panalytical Ltd, Malvern, UK). Such arrangement enabled rapid monitoring of airborne droplets shooting off the hot surface. Then the liquid suspension was supplied to the hot surface by an air displacement micropipette (Eppendorf South Pacific Pty. Ltd., North Ryde, NSW, Australia) similarly to our previously reported procedure^11^. A narrow range of temperatures in the film boiling regime was used, as it was previously proven to be the most efficient for droplet splashing and rebound.

#### Bacterial strains

Two common environmental bacterial strains obtained from Southern Biological (Nunawading, VIC, Australia); ATCC 6633 Gram-positive Bacillus subtilis (*B. subtilis*) and ATCC 27325 Gram-negative Escherichia coli (*E. coli*) were used throughout this study. *E. coli* bacterial cells are known to be sensitive to pasteurization if present in a liquid material. 99.99% reduction of the population can be achieved in less than one minute of pasteurization at the temperature of 62 °C^15^. *B. subtilis* vegetative cells and spores are characterized by higher heat resistance and are capable to survive at the temperature of 55 °C for a prolonged period of time^16^.

#### Bacterial culturing

A detail procedure of bacterial culturing could be found in [11]. In brief, microbial strains *B. subtilis* and *E. coli* were added into nutrient broth solution (OXOID Ltd., Basingstoke, Hampshire, England), prepared with concentration of 1.3 g per 100 ml of deionised and sterilised water, and placed in an incubator with the temperature of 37 °C for 18 hours. On completion of the incubation procedure, an aliquot of the microbial suspension was acquired prior to each experimental run for determination of culturable bacteria concentration by following plating technique. A sample of 0.1 mL of an appropriate 10-fold suspension dilution was applied onto the Nutrient Agar (NA) plate and carefully spread across the surface. Then, the plates were incubated over 24 hours at the temperature of 37 °C. A colony counter (Biolab, Clayton, VIC, Australia) was then used to count colonies and the corresponding microbial concentration in the in the liquid was determined and expressed in colony forming units (CFU) per mL of microbial suspension. To minimize/eliminate sporulation of *B. subtilis* cells due to extended shelf life, a fresh microbial suspension was prepared for each experimental run. The original microbial suspension as well as ten-fold diluted solutions were used in experiments upon necessity.

#### Experimental setup

A conical flask with 25 ml of deionised and sterilised water and a magnetic stirring bar was placed on a stirring hot plate with controllable temperature (Model PC-420D, Labnet International Inc., Edison, NJ, USA). The water was heated, and the temperature of water was monitored by calibrated K-type thermocouple operated by digital dual input thermometer (Model TM-1300K, Protek, Allendale, NJ, USA).

When the water temperature reached 100 °C, one millilitre of microbial suspension containing a certain concentration of bacteria was added into the water at continuous agitation. 75 ml of deionised and sterilised water were gradually added into the flask to decrease the temperature of the liquid and create favourable conditions for the survival of microorganisms. In order to evaluate the influence of the cooling time (length of cooling period) on survival of microorganisms the cold water was added with four different periods of time in a range from 0 to 15 seconds.

The liquid was removed from the hot plate immediately after mixing and the concentration of culturable microorganisms was measured in 0.1 ml of the liquid with the method described above. The results were analysed taking into account the additional 100 ml of water mixed with the bacterial suspension.

## Results and Discussion

**Figure 2:**
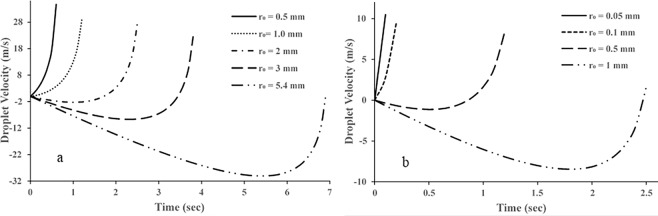
Influence of initial droplet radius on droplet lifetime and velocity at the wall temperature of 673 K and vapour layer thickness of (**a**) 1 µm and (**b**) 2 µm.

Droplet velocity as a function of time is demonstrated in Fig. 2. As it can be observed from the graphs, the velocity may be negative under certain conditions. This occurs with a high vapour layer thickness, large initial droplet size (radius) and small temperature gradient. The negative velocity means that the droplet stays on the surface during a particular time before departure if has not evaporated yet. The end of each curve shows the moment when the droplet totally evaporates.

**Figure 3:**
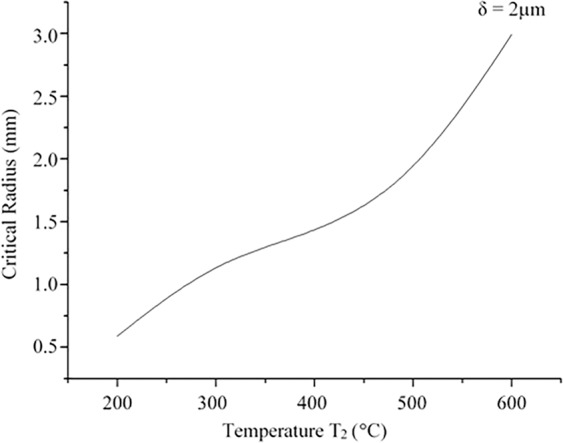
Critical radius of the droplet capable to rebound as a function of wall temperature at the vapour layer of 2 µm.

The critical radius of the droplet capable of shooting off is determined for the conditions when the beginning and the end of the velocity-time curve are at zero. Figure 3 demonstrates the largest radius of the droplet capable of departing from the surface as a function of the surface temperature if the vapour layer thickness is 2 μm. As it can be observed from the diagram the critical radius increases with the raising temperature.

**Figure 4:**
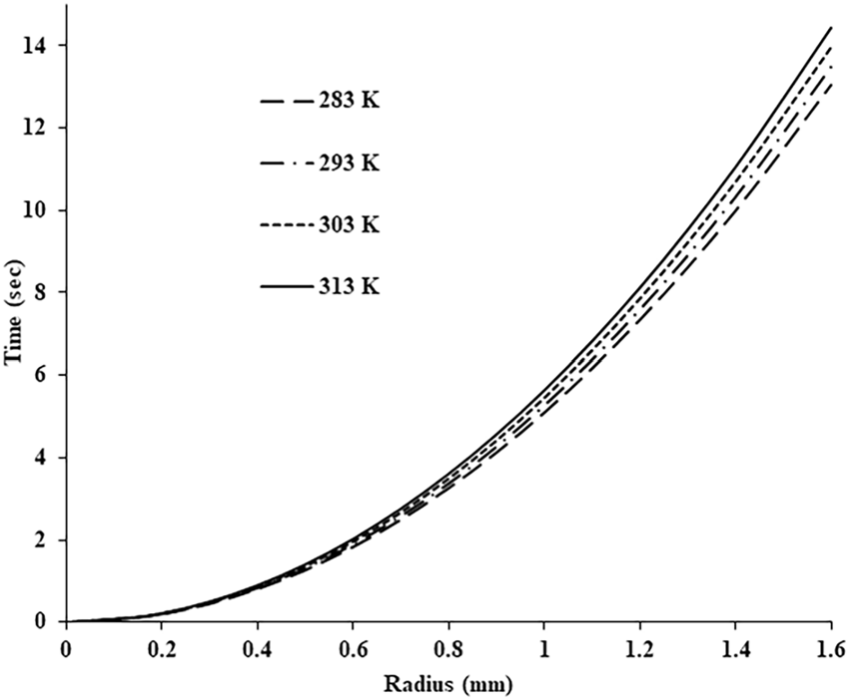
The relationship between the droplet radius and the time required to cool the droplet down to a microbial safe temperature of 40 °C.

The relationship between the droplet radius and the time required to cool the droplet down to the safe temperature of 40 °C is shown in Fig. 4. The diagram demonstrates that the cooling time mainly depends on the initial radius of the droplet while the influence of the ambient temperature is marginal.

The process of droplet departure in the film boiling regime has been extensively investigated with several models developed for the rebound regime using different boundary parameters along the interface between the liquid droplet and the vapour layer beneath.

When a low velocity droplet impacts a heated wall at high temperature, the droplet rebound is forced by pressure from the vapour layer. However, the numerical investigations of droplet rebound were developed either with or without a vapour layer model. In the second case the vapour flow is considered individually to determine the pressure and velocity of the vapour layer^17^.

Although surface temperature and drop velocity are the most important parameters influencing both the droplet behaviour and heat transfer^9^, this study does not consider the influence of droplet velocity and is focused on the droplet behaviour driven by the vapour layer. Ge and Fan^18^ demonstrated the accumulation of the kinetic enargy of the droplet in the post impact droplet spreading process and its conversion to surface energy that leads to the droplet departure. However, the residence time of the droplet on solid surface was shown almost independent of the impact velocity.

Celestini and colleagues^19^ investigated theoretically and experimentally the final stage of the droplet evaporation and demonstrated that under certain conditions the droplets suddenly take-off and are capable of reaching an elevation which is much higher than their radius. Heat transfer in a drop collision onto a heated surface depends on the difference between the surface temperature and the liquid saturation temperature (boiling point). When the wall temperature is significantly higher than the saturation temperature of the droplet and exceeds the Leidenfrost temperature, a thin vapour layer formed between the wall and the droplet decreases the contact and, therefore, reduces the heat removed from the wall^17^.

**Figure 5:**
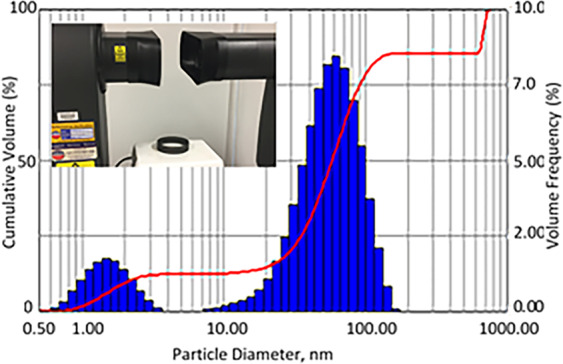
Droplet size departed from the hot surface (experimental setup is shown in inset).

Experimental results on the droplet size investigations are shown in Fig. 5. As is seen, strategic location of the heating element underneath Spraytec laser diffraction instrument enabled most of generated droplets passing through a measuring laser module enabling dynamic monitoring of airborne droplets. Two-modal size distribution, demonstrated in Fig. 5, was detected in all the 15 experimental runs with minor variations (<15%) in maximum droplet size for each mode. The shape of the graph is simply explained by two main mechanisms of the droplet generation; (1) condensation of vapour resulting in the fine droplet production, and (2) droplet take off resulting from liquid/hot surface interaction. The second mechanism is considered to be responsible for the particle generation with sizes of almost 1 mm. The experimental outcome justifies theoretical results shown previously in Fig. 3 where the maximum droplet size at this temperature can be up to 1.5 mm.

**Figure 6:**
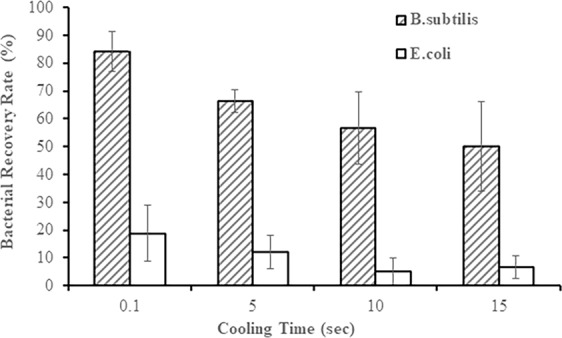
Survivability of bacteria as a function of cooling time (error bars represent standard deviation of at least 3 measurements).

Figure 6 shows the correlation between the survival of bacteria and cooling time. The shortest cooling time was associated with the highest survival rates. Overall *B. subtilis* characterized with higher survivability compared to *E. coli* that also confirms the results obtained in the previous research. It should be noticed that for the shortest time period of 0.l sec, 20% of *E. coli* and almost 90% of *B. subtilis* survived after the process. The survival of both microorganisms was decreasing with the increase of time reaching approximately 7% and 50% for *E. coli* and *B. subtilis* respectively over the longest realistic duration of the cooling process of 15 seconds. It ought to be noticed that further increase of the experiment duration would not be required, as 15 seconds is sufficient time for all hot droplets, even those with the maximum possible diameter, to be cooled down to 40 °C, which is safe for the most of bacterial strains.

These results can be compared against outcomes previously reported by Mirskaya and Agranovski^11^, where the microbial recovery rates of *E. Coli* and *B. Subtilis* were directly measured in the airborne droplets departed from hot surfaces at the levels of 1% and 4% for two strains respectively. The results of the previous study demonstrated up to 10 times lower recovery rates than the results obtained in the current project. A simple explanation of such discrepancy is based on the fact that the experiment in the current project does not involve any material losses of aerosolised microbial suspension. Such losses are unavoidable in the experiment used in [11] where a substantial amount of microbial material physically settles on the walls of the experimental setup and connecting pipelines and remain unaccounted. For comparison, experiments evaluating losses related to microbial aerosolization in nebulizers show that the losses can reach around three orders of magnitude, even for robust microorganisms (vaccinia virus), due to particle losses and microbial inactivation^20^. The outcome shows that the losses on hot surfaces are even lower as compared to losses during nebulizer operation. In any case, the amount of bioaerosols generated in interaction of microbial suspension with a hot surface can be substantial and requires appropriate actions to minimise health and environmental damage effects.

There are a few very important outcomes ought to be considered. First, taking into account that many industries use cooling water from local reservoirs, it can become a source of aerosolization of pathogenic microorganisms, which are not common in the ambient air and can cause serious health issues; for example, Legionella strains. Second, due to evaporation of aerosolized droplets, most of liquid will be lost. The remaining droplet can become quite small, almost reaching the size of bacterial diameter, and capable of travelling over long distances before final settlement to the ground. However, the minimal size of airborne particle would be dictated by the size in microbial particle Finally, in contrast with industrial environment, many residents would not be aware of the fact, that water from local reservoirs can be potentially contaminated and be dangerous for use in domestic applications, including saunas, extinguishing of open fires, cooling of outdoor equipment (BBQ, hot smoking houses, etc). Some additional instructions in the operational manuals are important for minimizing any potential risks associated with the processes.

## Conclusions

1. Numerical investigation demonstrated that the ability of a droplet to shoot off a hot surface depends on the surface temperature and the droplet size. The aerosolised droplet size increases from 0.5 to 3 mm for the surface temperature rise from 200 to 600 °C respectively.
2. The critical radius of the droplet capable of departing from the hot surface was estimated and verified experimentally. Depending on the experimental conditions it can reach a few millimetres in diameter.
3. The droplet cooling time mainly depends on the initial droplet radius while the influence of the ambient temperature is marginal.
4. The shortest cooling time is characterised with the highest survival rates of common bacterial strains *B. subtilis* and *E. coli*. However, even the numerically obtained realistic cooling time of 15 seconds did not ensure complete bacterial inactivation in the liquid.
